# Progressive Unilateral Cavitary Lung Disease With Nondiagnostic Bronchoalveolar Lavage Flow Cytometry Mimicking Sarcoidosis: A Case Report

**DOI:** 10.7759/cureus.102523

**Published:** 2026-01-28

**Authors:** Ikken Aisin

**Affiliations:** 1 Family and Community Medicine, University of California San Francisco School of Medicine, Fresno, USA

**Keywords:** bronchoalveolar lavage, cavitary lung disease, computed tomography, differential diagnosis, flow cytometry, lung mass, mediastinal lymphadenopathy, sarcoidosis

## Abstract

Bronchoalveolar lavage (BAL) cellular analysis is frequently used as an adjunct in the evaluation of interstitial lung disease (ILD), including sarcoidosis. Findings such as lymphocyte fraction and CD4/CD8 ratio may be supportive in the appropriate context but are neither specific nor diagnostic. We report the case of a 41‑year‑old woman with progressive unilateral cavitary lung disease and mediastinal lymphadenopathy initially raising concern for sarcoidosis. BAL flow cytometry from the affected lung was non‑diagnostic due to poor cell viability and near absence of T‑cell populations. Serial computed tomography (CT) imaging demonstrated features atypical for sarcoidosis, including bronchial cutoff, cavitation, and contralateral pulmonary nodules. This case highlights the limitations of BAL flow cytometry and reinforces the importance of multidisciplinary diagnostic integration in complex pulmonary disease.

## Introduction

Cavitary lung lesions and lung masses present a broad diagnostic challenge, encompassing infectious, malignant, and inflammatory etiologies. Pulmonary sarcoidosis, infection, and malignancy often overlap in both clinical presentation and imaging appearance, complicating early diagnostic assessment. Central lung tumors may cause airway obstruction with post‑obstructive consolidation or cavitation, while infectious and granulomatous diseases may present as mass‑like opacities, resulting in substantial radiographic overlap and diagnostic uncertainty [1].

Patients with cavitary pulmonary lesions may present with nonspecific respiratory symptoms or may be incidentally discovered on imaging. The differential diagnosis is broad and includes primary lung malignancy, metastatic disease, granulomatous infections such as tuberculosis (TB) or nontuberculous mycobacterial disease, fungal infections, and noninfectious inflammatory conditions, including sarcoidosis and vasculitis. Unilateral involvement, airway obstruction, cavitation, and associated lymphadenopathy may raise concern for serious underlying pathology, yet these features are not disease‑specific and require careful integration of clinical, radiologic, and pathologic data.

Bronchoscopy with bronchoalveolar lavage (BAL) is commonly incorporated into the evaluation of suspected interstitial and inflammatory lung disease. In selected clinical contexts, BAL lymphocyte fraction and CD4/CD8 ratio may increase diagnostic confidence for sarcoidosis [2]; however, these parameters are neither sensitive nor specific and must be interpreted alongside imaging findings and clinical evolution [3]. BAL cellular analysis is inherently adjunctive, and results may be limited by poor specimen quality, low cellularity, or reduced cell viability. Flow cytometry, while useful in select pulmonary and hematologic settings, may yield non‑diagnostic results when specimen adequacy is compromised. Reliance on BAL findings alone, particularly when technical limitations are present, may lead to diagnostic delay or misclassification [4].

Management strategies for cavitary lung lesions depend on the underlying etiology and may include antimicrobial therapy, oncologic evaluation, or invasive tissue sampling. Complications of delayed or incorrect diagnosis include disease progression, inappropriate treatment, and missed opportunities for timely intervention. This case illustrates a common but under‑recognized diagnostic pitfall: over‑interpretation of non‑diagnostic BAL flow cytometry in the setting of radiologic features that should prompt consideration of alternative diagnoses.

## Case presentation

A 41‑year‑old woman with no known history of immunodeficiency was referred for evaluation of progressive left‑sided pulmonary abnormalities identified on chest imaging. She had no prior diagnosis of sarcoidosis, TB, or malignancy at the time of presentation.

**Figure 1:**
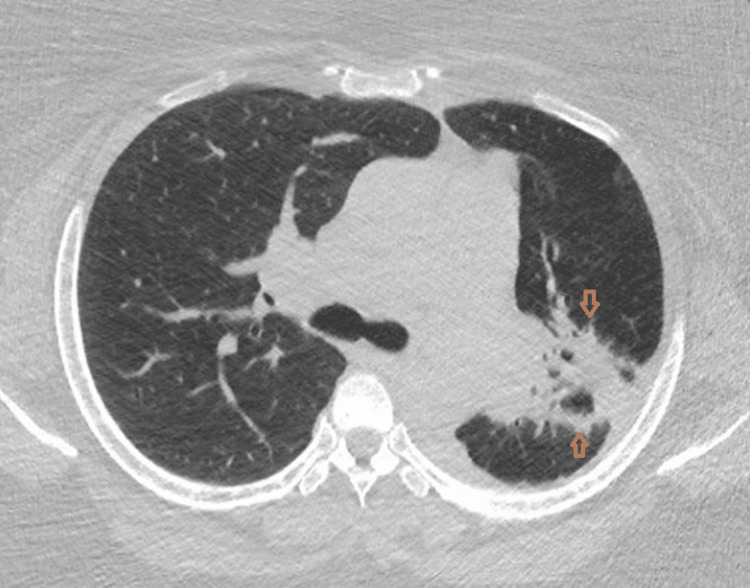
Axial lung window CT image showing dense left upper lobe consolidation with air bronchograms and ground-glass opacity abutting the mediastinum on the initial CT angiogram (arrows).

Initial CT angiography demonstrated extensive consolidation involving the left upper lobe and the superior segment of the left lower lobe, accompanied by mediastinal and hilar lymphadenopathy. Over time, serial imaging showed progressive structural distortion, including bronchial narrowing, volume loss, and eventual cavitary transformation (Figure 1).

The patient was a nonsmoker with no known occupational exposure to asbestos, silica, metal dusts, or organic antigens. There was no history of TB exposure, incarceration, homelessness, or recent travel to TB‑endemic regions. She was not immunosuppressed and had no known autoimmune disease.

Flexible bronchoscopy with BAL was performed, sampling the left upper lobe. Flow cytometry was conducted at Integrated Oncology. The specimen demonstrated poor cell viability and low cellular yield, significantly limiting interpretation. Immunophenotyping revealed virtually absent CD2‑positive and CD3‑positive T‑cell populations, and the CD4/CD8 ratio was not evaluable. Given the limited cellularity, these findings were considered non‑diagnostic and interpreted with caution.

As part of the infectious evaluation, sputum and blood studies were obtained. Sputum studies, including bacterial culture, acid‑fast bacilli smear, and fungal testing, were negative. Blood cultures showed no growth, and routine laboratory testing did not demonstrate leukocytosis or other findings suggestive of systemic infection. Serologic and microbiologic testing did not identify an infectious etiology, and results were interpreted in conjunction with imaging and clinical progression rather than in isolation.

**Figure 2:**
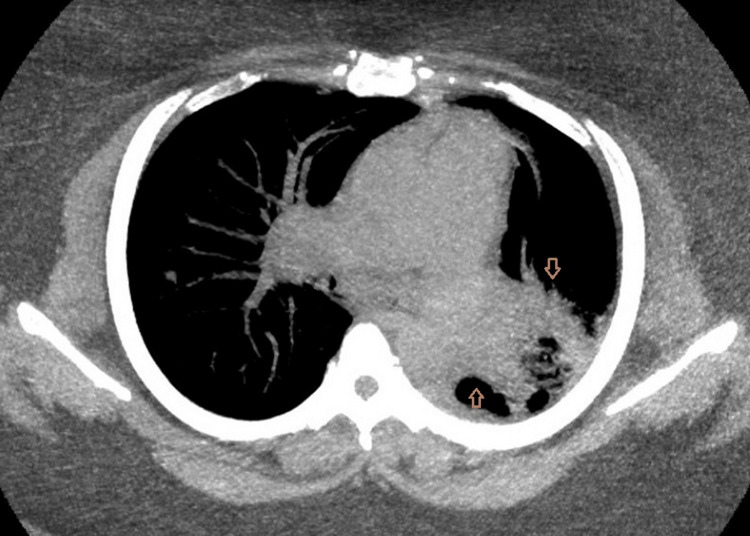
Axial soft tissue window CT image demonstrating heterogeneous left-sided consolidation with expansion and internal gas pockets, along with mild rightward mediastinal shift, concerning for necrotic consolidation (arrows).

**Figure 3:**
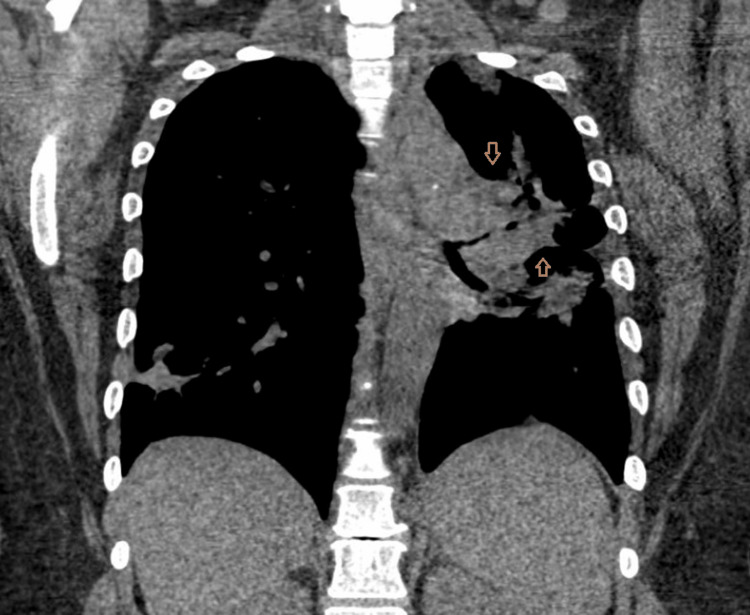
Coronal CT reconstruction demonstrating left-sided cavitary lesions with lung volume loss and multiple contralateral right-sided pulmonary nodules (arrows).

Follow-up CT performed on January 17, 2023, demonstrated expanded mediastinal and hilar lymphadenopathy, including a subcarinal lymph node measuring 15 × 28 mm. New cavitary lesions were identified within the left lung, measuring 23 × 32 × 20 mm in the left upper lobe and 21 × 48 × 33 mm in the superior segment of the left lower lobe. In addition, multiple new and enlarging right‑sided pulmonary nodules were noted, the largest measuring 8 mm, along with new peripheral consolidations (Figures 2-3).

The patient was subsequently lost to follow‑up after interval imaging demonstrated disease progression. As a result, the final etiology remains uncertain, highlighting the challenges posed by incomplete follow‑up in patients with concerning but non-diagnostic initial evaluations.

## Discussion

BAL cellular analysis can provide supportive information in selected clinical settings, but its limitations are well recognized. BAL lymphocyte fraction and the CD4/CD8 ratio are not diagnostic for sarcoidosis, as substantial overlap exists with other interstitial lung diseases and infectious processes. Although sarcoidosis may be associated with a higher CD4/CD8 ratio, similar findings can occur in other conditions, while some infections, such as TB, may also demonstrate elevated ratios. Overall, the BAL CD4/CD8 ratio has modest diagnostic performance for sarcoidosis, with reported sensitivity of approximately 53-59% and specificity of 83-92% when a cutoff of 3.5 is used. Diagnostic accuracy varies with pretest probability, disease stage, and patient population. Accordingly, an elevated ratio may increase diagnostic confidence in the appropriate clinical and radiographic context, but normal or low ratios do not exclude sarcoidosis, and false positives can occur [3]. Clinical guidelines emphasize that BAL findings should be interpreted as adjunctive data and not relied upon in isolation when establishing a diagnosis.

In this case, BAL flow cytometry was further compromised by poor specimen quality and near absence of evaluable T-cell populations [5], rendering the results non‑diagnostic. Under such circumstances, BAL findings should not override discordant imaging features. The radiologic pattern observed, including unilateral hilar mass effect, bronchial cutoff, cavitation, and contralateral nodules, is atypical for sarcoidosis and more concerning for malignancy or chronic infection [1]. These findings warranted continued diagnostic evaluation despite equivocal ancillary testing.

The differential diagnosis in this clinical context included primary lung malignancy, lymphoma, chronic infectious etiologies such as TB or fungal disease, and granulomatous inflammatory conditions, including sarcoidosis. Negative sputum and blood studies reduced concern for acute systemic infection but did not exclude indolent or localized infectious processes. Importantly, the patient was lost to follow‑up after the interval imaging demonstrated disease progression, and definitive tissue diagnosis was therefore not obtained. As a result, the ultimate etiology and subsequent clinical course remain unknown.

This case highlights a core diagnostic principle in pulmonary medicine: no single test should be interpreted in isolation [3]. Multidisciplinary discussion integrating clinical history, imaging, BAL results, and, when necessary, tissue sampling remains essential to avoid premature diagnostic closure. Contrast-enhanced chest imaging was not obtained during the initial evaluation, which may have further characterized mediastinal and hilar lymphadenopathy; however, non-contrast CT was sufficient to identify mass‑like pulmonary opacities that required invasive diagnostic assessment. This limitation further underscores the importance of clinical judgment and recognition of the constraints of adjunctive diagnostic tests when results are non‑diagnostic, particularly in patients who may be lost to follow-up.

## Conclusions

This case highlights the limitations of BAL flow cytometry when interpreted without an appropriate clinical and radiologic context. Non-diagnostic BAL findings, including indeterminate cellular analysis and CD4/CD8 ratios with known limited sensitivity and specificity for sarcoidosis, should not provide false reassurance in the presence of imaging features concerning for malignancy or chronic infection. Progressive unilateral cavitary lung disease with airway obstruction and contralateral nodularity warrants a broad differential diagnosis and prompt multidisciplinary evaluation, with emphasis on tissue sampling when ancillary tests are inconclusive. Loss to follow-up in this case precluded definitive diagnosis and assessment of disease progression, underscoring the importance of coordinated follow‑up and cautious interpretation of adjunctive diagnostic tests to avoid premature diagnostic closure.
